# The role of BET epigenetic readers in acute myeloid leukaemia and other haematological malignancies

**DOI:** 10.1186/1868-7083-5-S1-S6

**Published:** 2013-08-19

**Authors:** Brian Huntly

**Affiliations:** 1 Cambridge Institute for Medical Research, Wellcome Trust/MRC Building, Cambridge, CB2 0XY, UK

## 

Acute myeloid leukaemia (AML) is an aggressive haematological malignancy with a dismal long-term survival. Novel therapeutic agents are therefore urgently required to improve outcomes. AML is highly heterogeneous at the molecular, cellular and clinical level, however common themes have recently emerged, including transcriptional dysregulation and alteration of epigenetic control. Indeed, deep sequencing efforts have outlined recurrent mutations in a number of epigenetic regulators in AML. This observation has potential clinical implications for, whilst the genome is fixed, the epigenome is dynamic, making it a potentially attractive target for therapeutics. This talk will outline the clinical and biological background to AML and will also provide context for targeting epigenetic readers.

The bromodomain and extra-terminal (BET) proteins are a family of bromodomain-containing epigenetic readers. They bind to acetylated lysines in histones and other proteins and interact with a number of transcriptional regulators during transcriptional commitment, initiation and elongation. Recently, inhibitors of the protein-protein interaction between BET proteins and acetylated lysines have been developed, initially as anti-inflammatory immunomodulators. We will outline our rationale for utilising these inhibitors in a highly specific form of acute leukaemia, mixed lineage leukaemia (MLL)-fusion leukaemias, and will briefly show published data justifying this hypothesis. These data will outline the mechanism of action of these inhibitors, demonstrating the removal from chromatin of BET proteins and interacting transcriptional complexes, leading to downregulation of transcription at loci critical for leukaemogenesis. Finally, preclinical studies demonstrating the efficacy of these inhibitors in murine models, cellular models and patient samples of MLL-fusion leukaemia will outline the efficacy and clinical promise of these agents for this aggressive form of leukaemia.

The majority of the talk will focus on unpublished data investigating the extension of I-BET therapy to other subtypes of AML. We will demonstrate that many other subtypes of AML are sensitive to growth inhibition with I-BET. This sensitivity appears to be due to the downregulation of a common transcriptional programme across a number different AML subtypes. We will show data predicting that this programme, along with specific recurrent mutations, will form the basis of predictive biomarkers of sensitivity and response to I-BET therapy, thus informing clinical trials. We next focused our attention on AML with mutations of the nucleophosmin (NPM1) gene and describe the mechanism underpinning the profound sensitivity of this common AML subtype. Finally, we will show evidence of preclinical efficacy in murine, cellular and primary patient material models of NPM1c AML.

At the end of the talk I will attempt to put these findings into clinical context, briefly summarising the results of BET inhibition in other haematological malignancies and discussing an outline of plans for a phase I clinical trail of BET inhibition in relapsed haem malignancies to be carried out within the UK.

